# Therapeutic approaches to sinonasal NUT carcinoma: a systematic review

**DOI:** 10.1007/s00405-024-08489-0

**Published:** 2024-02-08

**Authors:** Anastasia Urbanelli, Letizia Nitro, Carlotta Pipolo, Alberto Maccari, Andrea Albera, Gian Luca Fadda, Giovanni Felisati, Roberto Albera, Giancarlo Pecorari, Emanuela Fuccillo, Alberto Maria Saibene

**Affiliations:** 1 Department of Surgical Sciences, Otorhinolaryngology Unit, University of Turin, Via G. Verdi, 8, 10124 Turin, Italy; 2 Department of Health Sciences, Otorhinolaryngology Unit, Santi Paolo e Carlo Hospital, University of Milan, Milan, Italy; 3 Otorhinolaryngology Unit, San Luigi Gonzaga University Hospital, University of Turin, Turin, Italy

**Keywords:** Sinonasal NUT carcinoma, Nuclear protein in testis carcinoma, NUT midline carcinoma, Sinonasal malignancy, Head and neck tumors

## Abstract

**Purpose:**

Sinonasal nuclear protein in testis carcinoma (SNUTC) is a rare, aggressive malignancy caused by genetic rearrangements in the NUTM1 gene. The prognosis of SNUTC ranks among the most unfavorable within the naso-sinusal district, with an overall survival of 9.7 months. This systematic review aimed to determine the best therapeutic strategy for SNUTC.

**Methods:**

We reviewed eligible articles for patient demographics, TNM and stage at presentation, best response after primary treatment, disease-free survival and overall survival (OS) times, other following therapy lines, and final outcomes.

**Results:**

Among 472 unique citations, 17 studies were considered eligible, with reported treatment data for 25 patients. Most studies (*n* = 12) were case reports. The most frequently administered treatment regimen was surgery as primary treatment and combined radiochemotherapy as second-line or adjuvant treatment. Four patients were alive at follow-up.

**Conclusion:**

Basing on the existing literature, a standardized line in the treatment of SNUTC is not yet well delineated. A self-personalized strategy of therapy should be drawn on each patient affected by SNUTC.

## Introduction

Nuclear protein in testis (NUT) carcinomas are rare, highly aggressive malignancies, caused by genetic rearrangements in the NUTM1 gene. They generally arise in midline structures of head and neck or thorax, but every organ can be virtually affected. The most frequent mutation involves the translocation of the NUTM1 gene to form a fusion protein with the BRD4 gene on chromosome 19p13.1 [1]. Other translocation variants involve the fusion of the NUTM1 gene on 15q14 with another gene such as BRD3 on 9q34.2 [2]. From a histological perspective, these tumors generally show non-specific features, ranging from poorly differentiated carcinomas to carcinomas with prominent squamous differentiation. For this reason, their morphological diagnosis is often difficult and must be integrated with molecular methods to demonstrate a rearrangement of the NUTM1 gene (i.e., fluorescent in situ hybridization—FISH) [1, 3].

Primary SNUTC (sinonasal NUT carcinomas) of the nasal cavity and paranasal sinuses are extremely rare, causing diagnostic and therapeutic difficulties. Presenting symptoms are usually non-specific and represented by nasal obstruction, rhinorrhea, epistaxis, acute sinusitis with or without diplopia, exophthalmos, and proptosis [4, 5]. Therefore, SNUTCs are often confused with infection processes or with benign nasal conditions, leading to delayed diagnosis. Given the rarity of the SNUTC, its frequent diagnostic delay due to the lack of characteristic clinical and histopathological features and its poor diagnosis (with a median overall survival of 9.7 months [6]), recommended treatments have not been established yet.

This review aims to summarize and analyze the currently available therapeutic strategies for nonmetastatic SNUTC in terms of disease-specific survival and disease-free survival.

## Materials and methods

### Search strategy

After registering with the PROSPERO database (ID CRD42023390827), we conducted a systematic review between January 11, 2023, and May 3, 2023, according to PRISMA reporting guidelines [7]. Systematic electronic searches were carried out in English, Italian, German, French, and Spanish, for articles reporting original data on therapeutic strategies for SNUTC.

On January 11, 2023, a primary search was performed on the MEDLINE, Embase, Web of Science, Cochrane Library, Scopus, and ClinicalTrials.gov databases combining the terms “(NUT OR NUT carcinoma OR NUT midline carcinoma) AND (nose OR sinus OR maxillary OR frontal OR sphenoid OR ethmoid)”. Complete search strategies and the number of items retrieved from each database are provided in Table 1. The references of selected publications were then examined to identify further reports that were not found by database searching, and the same selection criteria were applied.

**Table 1:** Search strategy details and items retrieved from each consulted database

Database	Search date	Query	Items retrieved (n)
Medline	January, the 11th, 2023	(("nuts"[MeSH Terms] OR "nuts"[All Fields] OR "nut"[All Fields] OR "NUT carcinoma"[All Fields] OR "NUT midline carcinoma"[All Fields]) AND ("nose"[MeSH Terms] OR "nose"[All Fields])) OR ("paranasal sinuses"[MeSH Terms] OR ("paranasal"[All Fields] AND "sinuses"[All Fields]) OR "paranasal sinuses"[All Fields] OR "sinus"[All Fields] OR "sinus s"[All Fields]) OR ("maxilla"[MeSH Terms] OR "maxilla"[All Fields] OR "maxillary"[All Fields] OR "maxillaries"[All Fields] OR "maxillaris"[All Fields]) OR ("frontal"[All Fields] OR "frontales"[All Fields] OR "frontalization"[All Fields] OR "frontally"[All Fields] OR "frontals"[All Fields]) OR ("sphenoid bone"[MeSH Terms] OR ("sphenoid"[All Fields] AND "bone"[All Fields]) OR "sphenoid bone"[All Fields] OR "sphenoid"[All Fields] OR "sphenoids"[All Fields] OR "sphenoidal"[All Fields] OR "sphenoiditis"[All Fields]) OR ("ethmoid bone"[MeSH Terms] OR ("ethmoid"[All Fields] AND "bone"[All Fields]) OR "ethmoid bone"[All Fields] OR "ethmoid"[All Fields] OR "ethmoids"[All Fields] OR "ethmoid sinusitis"[MeSH Terms] OR ("ethmoid"[All Fields] AND "sinusitis"[All Fields]) OR "ethmoid sinusitis"[All Fields] OR "ethmoiditis"[All Fields] OR "ethmoidal"[All Fields])	170
Embase	January, the 11th, 2023	(nut OR 'nut carcinoma' OR 'nut midline carcinoma') AND ('nose'/exp OR nose OR 'sinus'/exp OR sinus OR 'maxillary'/exp OR maxillary OR frontal OR 'sphenoid'/exp OR sphenoid OR 'ethmoid sinus'/exp OR 'ethmoid sinus')	464
Cochrane library	January, the 11th, 2023	NUT OR "NUT carcinoma" OR "NUT midline carcinoma") AND (nose OR sinus OR maxillary OR frontal OR sphenoid OR ethmoid in Title Abstract Keyword – (Word variations have been searched)	10
Web Of Science	January, the 11th, 2023	NUT OR "NUT carcinoma" OR "NUT midline carcinoma") AND (nose OR sinus OR maxillary OR frontal OR sphenoid OR ethmoid (all fields)	50
Clinicaltrials.gov	January, the 11th, 2023	(NUT OR "NUT carcinoma" OR "NUT midline carcinoma") AND (nose OR sinus OR maxillary OR frontal OR sphenoid OR ethmoid)	17
Scopus	January, the 11th, 2023	TITLE-ABS-KEY (NUT OR "NUT carcinoma" OR "NUT midline carcinoma") AND (nose OR sinus OR maxillary OR frontal OR sphenoid OR ethmoid)	283
Total non unique hits			994

We included all article types excluding meta-analyses and systematic or narrative reviews, which were nevertheless hand-checked for additional potentially relevant papers. Exclusion criteria were as follows: non-human studies, papers carried out in other languages than English, Italian, German, French, or Spanish, patients presenting NUT carcinomas of head and neck regions other than the sinonasal tract, and studies that reported follow-up periods of less than 12 months (unless the patient died within the year). No minimum study population was required. No publication date restriction was applied. Given the rarity of this neoplasm, we included only articles which stated that the diagnosis had been confirmed by molecular identification of the NUT gene.

Abstract and full texts were reviewed in duplicate by different authors. At the abstract review stage, we included all studies that were deemed eligible by at least one rater. At the full-text stage, disagreements were resolved by achieving consensus among raters.

### PICOS criteria

The Population, Intervention, Comparison, Outcomes, and Study (PICOS) framework for the review was defined as follows:

- P: all patients with a primary sinonasal NUT carcinoma (SNUTC)
- I: any kind of treatment for SNUTC, either surgical, radiotherapeutic, chemotherapeutic, or combined
- C: comparisons between different kinds of treatments and with no treatment
- O: disease-specific survival and disease-free survival
- S: original studies of any kind and clinical setting (except meta-analyses)

### Data extraction and quality assessment

For each article included, we recorded: study type, the overall number of patients included, female to male ratio, patients’ age at diagnosis, TNM and stage at presentation, prior therapy (any therapy carried out before the final diagnosis of SNUTC), primary treatment (i.e., surgery, chemotherapy, radiotherapy, combined therapy), adjuvant therapy (when performed), best treatment response (i.e., partial response, complete response, disease progression), disease-free survival (DFS) time in months, overall survival (OS) time in months, type of further therapy lines (second, third, etc.…) and respective progression-free survival, and final outcome (i.e., death from disease—DFD, alive with disease—AWD, alive without disease—AWOD). We excluded papers that reported follow-up periods of less than 12 months (unless the patient died within a year). Two authors extracted data and rated studies in duplicate, and disagreements were resolved by consensus.

Studies were assessed for both quality and methodological bias according to the National Heart, Lung, and Blood Institute Study Quality Assessment Tools (NHI-SQUAT) [8] for case series and cohort studies and the Joanna Briggs Institute Critical Appraisal tools (JBI-CAT) for case reports [9]. With the same methodology adopted for systematic reviews with middle-to-low evidence levels in comparable recent reviews [10], items were rated as “good” if they fulfilled at least 80% of the items reported in the JBI-CAT or NHI-SQUAT, “fair” if they fulfilled between 50 and 80% of the items, and “poor” if they fulfilled less than 50% of the items, respectively.

The level of evidence for clinical studies was scored according to the Oxford Centre for Evidence-based Medicine (OCEBM) level of evidence guide [11].

Due to the considerable heterogeneity of study populations, study methods, and the predominantly qualitative nature of collected data, no initial meta-analysis was planned or performed a posteriori.

## Results

**Fig. 1:**
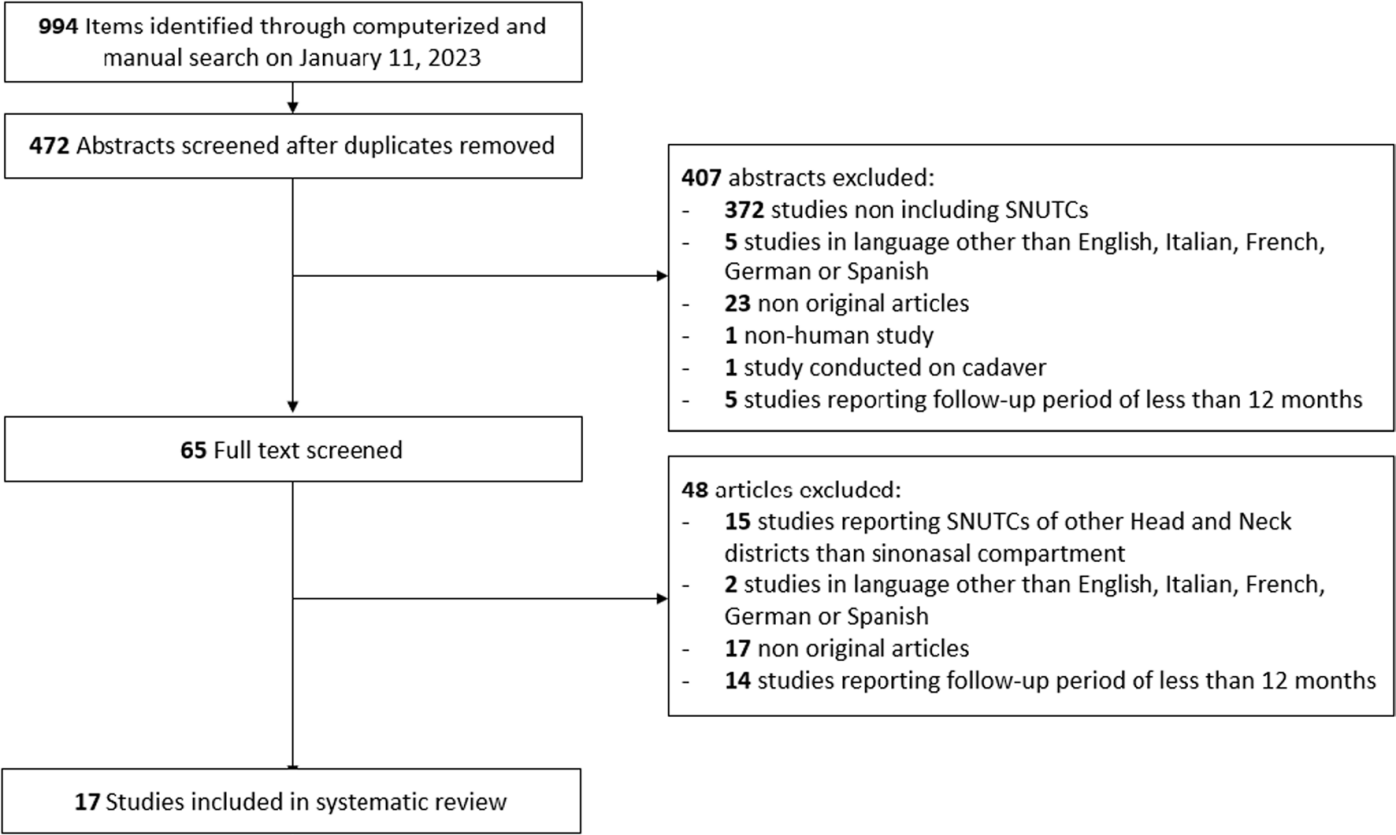
PRISMA style flow diagram of studies through systematic review

Among the 472 unique research items initially identified, 65 published reports were selected for full-text evaluation. No further report was identified from full-text evaluation after reference checking. Overall, 17 studies published between 2011 and 2022 were retained for analysis (Fig. 1) [3, 4, 12–26].

5 articles were case series, and the remaining 12 articles were case reports. All articles had a level of evidence IV according to the OCEBM scale. Articles were rated as good (*n* = 13), fair (*n* = 3), or poor (*n* = 1) according to NHI-SQAT or JBI-CAT tools. No significant biases toward the objectives of our systematic review were identified, while most articles lacked ample information allowing for patient comparison. Table 2 reports the study type, evidence, and quality rating for all studies included.

**Table 2:** Type of study, and evidence and quality rating of reviewed articles

Reference	Study type	OCEBM rating	Quality rating
Abreu et al., 2022 [12]	CS	4	G
Arimizu et al., 2018 [13]	CR	4	G
Bishop and Westra, 2012 [14]	CS	4	G
Chan et al., 2018 [15]	CR	4	P
Crocetta et al., 2021 [16]	CR	4	G
Davis et al., 2011 [3]	CR	4	F
Elkhatib et al., 2019 [4]	CR	4	G
Huang et al., 2022 [17]	CR	4	G
Klijanienko et al., 2016 [18]	CS	4	F
Laco et al., 2018 [19]	CS	4	F
Lee et al., 2019 [20]	CS	4	G
Maloley et al., 2019 [21]	CR	4	G
Muramatsu et al., 2022 [22]	CR	4	G
Oliveira et al., 2019 [23]	CR	4	G
Patel et al., 2021 [24]	CR	4	G
Stirnweiss et al., 2015 [25]	CR	4	G
Tosic et al., 2022 [26]	CR	4	G

*CS* case series, *CR* case report, *OCEBM* Oxford Centre for Evidence Based Medicine, *G* good, *P* poor, *F* fair

The 17 included studies had 25 participants whose ages at diagnosis ranged from 14 to 65 years (median 46, interquartile range 12). Patients were more frequently male (15 male vs. 9 female), while one paper did not report gender information. TNM and stage at presentation were reported only in a few studies. In the remaining cases, TNM and stage were derived from the description of the extent of the malignancy or from the provided radiological images. In particular, stages IV and III were the most common (18 out of 25 patients), both stage II and stage I were found only in 1 case, and for 5 patients the initial stage could not be assessed. Table 3 shows the demographic and clinical information for the treated patients.

**Table 3:** Demographic and clinical information on the treated patients for all included studies

References	Treated patients (*n*)	Female: male ratio (*n*: *n*)	Patients’ age at diagnosis (years)	TNM at presentation	Stage at presentation
Abreu et al., 2022 [12]	2	1:1	37; 43	pT3NOMO; pT4bN0M0	III; IVb
Arimizu et al., 2018 [13]	1	0:1	49	cT3NxM1*	IVc*
Bishop and Westra, 2012 [14]	3	0:3	26; 33; 48	N/R	N/R
Chan et al., 2018 [15]	1	0:1	48	T4bNxMx*	IVb*
Crocetta et al., 2021 [16]	1	1:0	56	cT3N0M0*	III*
Davis et al., 2011 [3]	1	1:0	54	cT2N0Mx*	II*
Elkhatib et al., 2019 [4]	1	1:0	47	cT4bN0M0*	IVb*
Huang et al., 2022 [17]	1	1:0	58	cT4aNxMx*	IVa*
Klijanienko et al., 2016 [18]	1	0:1	20	N/R	N/R
Laco et al., 2018 [19]	3	0:3	65; 46; 60	pT1N0M0; pT3N0M0; pT4aN0M0 (all cN0M0)	I; III; IVA
Lee et al., 2019 [20]	4	2:2	60; 45; 42; 29	N/R; pT4a; cT4bN + Mx; cT4bN0Mx	N/R; IVa; IVb; IVb
Maloley et al., 2019 [21]	1	1:0	47	cT4bNxMx*	IVb*
Muramatsu et al., 2022 [22]	1	0:1	18	cT4bN0M0*	IVb*
Oliveira et al., 2019 [23]	1	0:1	42	cT4bN0M0*	IVb*
Patel et al., 2021 [24]	1	0:1	39	cTxN2cM1*	IVc*
Stirnweiss et al., 2015 [25]	1	1:0	14	cT4aN0M0*	IVa
Tosic et al., 2022 [26]	1	N/R	47	cT4bNxM1*	IVc*

*N/R* not reported

^*^Derived from the description of the extent reported in the text

Before the final diagnosis of SNUTC, in 9 out of 25 patients a prior therapy consisting of endoscopic resection (*n* = 7) and chemotherapy (CT, *n* = 2) with vincristine/cyclophosphamide/doxorubicin or docetaxel/cisplatin/fluorouracil was carried out. Most patients (*n* = 11) underwent surgery as primary treatment, followed by combined chemoradiotherapy (CRT) (*n* = 6), exclusive radiotherapy (RT, *n* = 3), a combination of surgery and CT (*n* = 3), exclusive CT (*n* = 1), exclusive immunotherapy (IT) (*n* = 1), and combined IT + RT. Adjuvant therapy was administered in ten patients and consisted of exclusive CT, combined CT and RT, and exclusive RT (also extended to the thoracic and lumbar spine for palliative intent). After the failure of initial treatments, five patients in total received other than second-line therapies, which contemplated CT for four patients and CRT for one subject as third-line therapy, CT for two patients and IT for one patient as fourth-line therapy, and CT for one patient as fifth and sixth-line therapy. DFS and OS were extremely variable, ranging from 1 to 96 months and from 2 to 108 months, respectively. Table 4 shows the data on primary and following treatment approaches, the DFS and OS of each patient, and the final outcome at the end of follow-up time.

**Table 4:** Treatment regimens, DFS (disease-free survival), OS (overall survival) and final outcome for all patients

Reference	Treated patients (n)	Prior therapy	Primary treatment	Adjuvant therapy	Best treatment response	2nd line treatment	Progression free survival after 2nd line treatment (months)	DFS (months)	OS (months)	Outcome
Abreu et al., 2022 [12]	2	S (*n* = 2)	S + CT; S	C; none	CR (*n* = 2)	RT; CT	N/R (*n* = 2)	1.5; 1.4	14.9; 3.4	DFD (*N* = 2)
Arimizu et al., 2018 [13]	1	CT	CT	None	PR	CRT	None	0	9	DFD
Bishop and Westra, 2012 [14]	3	None	S (= 3)	CRT (*n* = 3)	PD (*n* = 3)	None (*n* = 3)	–	0 (*n* = 3)	8;11;16	DFD (*N* = 3)
Chan et al., 2018 [15]	1	None	RT	N/R	N/R	None	–	N/R	4	DFD
Crocetta et al., 2021 [16]	1	None	S	CRT	CR	None	–	1	6	DFD
Davis et al., 2011 [3]	1	None	CRT	None	PD	CRT	N/R	N/R	7	DFD
Elkhatib et al., 2019 [4]	1	S	RT	None	PD	CRT	2	0	5	DFD
Huang et al., 2022 [17]	1	None	S	None	PD	CRT	N/R	0	17	DFD
Klijanienko et al., 2016 [18]	1	None	S	CRT	CR	S	N/R	12	22	DFD
Laco et al., 2018 [19]	3	None	S (*n* = 3)	RT	CR; N/R; N/R	N/R (*n* = 3)	N/R	108; N/R; N/R	108; 8; 3	AWOD; DFD; DFD
Lee et al., 2019 [20]	4	None; S; none; none	RT; S + CT; CRT; S + CT	None	CR (*n* = 3); N/R	S; none; N/R; None	none (*n* = 4)	96; 8; 36; N/R	96; 36; N/R; N/R	DFD; AWOD; N/R; N/R
Maloley et al., 2019 [21]	1	S	CRT	None	PD	None	–	0	5	DFD
Muramatsu et al., 2022 [22]	1	S	CRT	None	CR	PBR	2 (end of follow-up)	9	18	AWOD
Oliveira et al., 2019 [23]	1	CT	CRT	None	PR	None	–	0	2	DFD
Patel et al., 2021 [24]	1	None	IT	None	PD	RT	N/R	0	21	AWD
Stirnweiss et al., 2015 [25]	1	None	S	None	PD	None	–	0	3	DFD
Tosic et al., 2022 [26]	1	S	IT + RT	RT	PR	None	–	0	4	DFD

*DFS* disease-free survival, *OS* overall survival, *S* surgery, *CT* exclusive chemotherapy, *RT* exclusive radiotherapy, *CRT* combined chemoradiotherapy, *IT* exclusive immunotherapy, *PBR* proton beam radiotherapy, *CR* complete response, *PR* partial response, *PD* progression of disease, *DFD* dead form disease, *AWOD* alive without disease, *AWD* alive with disease, *N/R* not reported

Concerning the treatment response, most patients (*n* = 9) showed complete response to primary therapy but only three of them were AWOD at the end of their follow-up period [19, 20, 22]. On the other hand, the primary treatment of nine patients resulted in a disease progression and three patients showed only partial response to the therapy. As shown in Table 4, 10 out of 25 patients underwent a second-line treatment which consisted of exclusive CT (*n* = 1), exclusive RT (*n* = 3), combined CT and RT (*n* = 4), and surgical treatment (*n* = 2). Of all patients who received second-line treatment, only one resulted in complete recovery. In particular, his first-line treatment consisted of CT (vincristine, doxorubicin, and cyclophosphamide, alternating with ifosfamide and etoposide) combined with RT, while as the second-line treatment he received proton beam radiotherapy (BPR) [22].

As for the final outcome at the end of the follow-up period, the majority (*n* = 19) were DFD, three patients were AWOD, and one was AWD. There is no outcome information for two patients.

## Discussion

To the best of our knowledge, this represents the first systematic review to analyze the currently available therapeutic approaches to treat SNUTC. Analyzing the existing literature, we found out only few eligible studies with a low level of evidence (case series and case reports); this demonstrates the huge variability upon this theme and the absence of a standardized therapy strategy for treatment of SNUTC. The lack of current guidelines on this topic is attributable to the rarity of the neoplasm and to its intrinsic aggressivity, to the unusual patient presentation, and to the rapid onset of symptoms that often occur when the disease is already in an advanced stage. All these factors contribute to SNUTC being a malignancy burdened with a diagnostic delay and an unfavorable prognosis. For all the reasons listed above, the current literature lacks randomized controlled trials. We identified only five case series and a few case reports (12 out of 17 selected works), which represent therefore only a starting point for building a standardized approach to this disease.

In total, 18 out of 25 patients in our review showed an advanced stage of SNUTC at the time of first diagnosis, which can be explained by at least 2 reasons. First of all, this late diagnosis could be a direct result of the poor differentiation characterizing SNUTCs, which are therefore characterized by high aggressiveness and by a dramatically rapid growth rate [27]. On the other hand, this might be due to the unspecific semeiology of SNUTCs. Presenting symptoms are varied and extremely non-specific, ranging from headache, rhinorrhea, and nasal obstruction to blurred vision, diplopia, and conjunctival chemosis. For this reason, it is not uncommon for this neoplasm to be treated initially as a benign condition (e.g., with antibiotics and systemic steroids), thus contributing to delaying proper treatment. In our review, only two patients presented with an early stage of SNUTC at diagnosis. In particular, a 54-year-old woman presented with a stage II neoplasm (cT2N0Mx) consisting in a 2.5 × 2 cm mass that involved her right nasal bone without evidence of regional or distant metastasis. She received intensity-modulated radiation therapy (IMRT) and concurrent CT (cisplatin) with no benefit on tumor growth and, subsequently, a second-line therapy based on CRT with ifosfamide/etoposide with concurrent RT, achieving only a partial response that ultimately resulted in the patient’s death only 7 months after diagnosis [3]. The second patient who presented in an early stage was a 65-year-old man with a cT1N0 SNUTC (stage I) arising in his nasal cavity. He was treated with radical surgical resection and with RT as adjuvant therapy with a complete response and without evidence of disease at the end of his follow-up period (which lasted for 108 months) [19].

High stage at diagnosis and aggressive behavior account for the dramatically poor prognosis of this condition, well-represented by collected data. If we consider the final outcome of treatment for patients included in this review, only three out of 25 patients were AWOD at the end of follow-up, and 1 patient was reported as AWD. Since our goal in this work is to provide the more appropriate therapeutic strategy for the treatment of SNUTC, a detailed treatment profile of each of these subjects is shown. In addition to the patient of Laco et al. already reported in the previous paragraph (who survived without disease for at least 108 months after surgery and adjuvant RT) [19], Lee et al. in their case series presented, among the other patients affected by the same malignancy, a 45-year-old woman which showed at her diagnosis a IVa SNUTC (pT4) of left ethmoid sinus initially treated with radical surgery for the suspicion of a cancerous change of inverted papilloma. After the right diagnosis of SNUTC, she received another radical surgical procedure consisting of a left anterior skull base craniofacial resection and a medial maxillectomy. She also received adjuvant concurrent CRT with cisplatin, obtaining a complete response without being reminded of a second-line therapy and showing an OS of 36 months [20]. Muramatsu et al. presented an 18-year-old woman with a SNUTC of the nasal cavity already in a IVb stage (cT4bN0) at diagnosis, treated with CRT based on vincristine, doxorubicin, cyclophosphamide plus RT (70 Gy/35 Fr) as primary treatment, which obtained complete response with a DFS of 9 months. She successively received second-line treatment with proton beam radiotherapy (PBR) due to a recurrence of the tumor in her left ethmoid sinus with skull base invasion. Her OS at the end of her follow-up period was 18 months without recurrence of the SNUTC [22]. Lastly, the only patient who resulted as AWD was a 39-year-old man who presented with a IVc SNUTC and, due to the high PD-L1 score on his specimen, consented to a clinical trial combining a PD-L1 checkpoint inhibitor and a Toll-like receptor 7 (TLR7) agonist, resulting in progression of disease at the end of the treatment. Therefore, he received RT as second-line therapy followed by CT with gemcitabine and paclitaxel (obtaining a progression-free survival of 9 months) and, lastly, he started another clinical trial based on a Bromodomain and extra terminal protein (BET) inhibitor. At the end of his follow-up period, he was AWD showing an OS of 21 months [24]. An important limitation of this review consists of the huge variability of the follow-up period of the included patients, which could lead to an important bias in the interpretation of the presented data. For example, the patient presented by Muramatsu et al. ended her follow-up period as AWOD after 18 months [22] but, on the other hand, the case reported by Klijanienko et al. showed a DFD outcome with a longer follow up-period and with a longer OS (22 months) [18]. This means that the length of the follow-up invariably affects the final outcome of the patients.

In conclusion, on the basis of our extrapolated data, some considerations are mandatory. First of all, even if most patients presented with an already advanced stage, an early stage (I or II) at presentation does not represent a guarantee of better success of the proposed therapy. In fact, of the two subjects which showed an early stage SNUTC, only one resulted as AWOD at his final outcome. Conversely, we detected other two patients who were AWOD despite presenting with a IVa and IVb stage, respectively. Moreover, among the three patients who showed no recurrence at the end of their follow-up period, we did not detect a similar strategy approach among them. In fact, they all received an individualized therapy scheme that varied with each other and that does not allow a common and standardized line of treatment to be defined. This may lead to the assertion that a self-personalized strategy of therapy should be drawn on each patient affected by SNUTC, which could be ideally based on the patient’s age and history, on the molecular characterization of his illness, and on the experience of the oncological team of each center.

## Data Availability

All data pertaining to this systematic review are available from the corresponding author upon reasonable request.
